# Correlations of Serum Lipid Parameters and Atherogenic Indices With Left Ventricular Diastolic Dysfunction Among Apparently Healthy Patients With Type 2 Diabetes Mellitus: A Multicenter In-Hospital Cross-Sectional Study

**DOI:** 10.1155/2024/4078281

**Published:** 2024-07-13

**Authors:** Yves Mayambu Dienda, Jean-Bosco Kasiam Lasi On'kin, Aliocha Nkodila Natuhoyila, Yves Lubenga, Tresor Mvunzi Swambulu, Jean-René M'buyamba-Kabangu, Benjamin Longo-Mbenza, Bernard Kianu Phanzu

**Affiliations:** ^1^ Cardiology Unit University Hospital of Kinshasa University of Kinshasa, Kinshasa, Democratic Republic of the Congo; ^2^ Unit of Endocrinology and Metabolism University Hospital of Kinshasa University of Kinshasa, Kinshasa, Democratic Republic of the Congo; ^3^ Department of Statistics Public Health School, Kinshasa, Democratic Republic of the Congo

## Abstract

**Background:** In adolescents with Type 1 diabetes, lipid ratios are predictors of left ventricular diastolic dysfunction (LVDD). However, whether this also applies to adults with Type 2 Diabetes Mellitus (T2DM) is unclear. This study is aimed at assessing the correlations of serum lipid parameters and atherogenic indices with LVDD in patients with T2DM.

**Methods:** This cross-sectional study included 203 patients with T2DM aged 59.9 ± 13.6 years (111 males, sex ratio: 1 : 2 in favor of males) from eight randomly selected urban hospitals. Demographic information was collected, an anthropometric assessment was performed, and blood pressure was measured. Fasting blood samples were obtained to assess total cholesterol (TC), high-density lipoprotein cholesterol (HDL-C), low-density lipoprotein cholesterol (LDL-C), triglycerides (TGs), glucose, and glycated hemoglobin. The atherogenic index of plasma (AIP), Castelli Risk Index I (CRI-I), Castelli Risk Index II (CRI-II), atherogenic coefficient, and non-HDL-C were determined using specific formulas. Diastolic function was assessed using echocardiography as per the 2016 updated guidelines of the American Society of Echocardiography (ASE) and the European Association of Cardiovascular Imaging (EACVI).

**Results:** Approximately 47.8% of the participants had LVDD. Compared with participants with normal diastolic function, those with LVDD were more likely to be older than 55 years (*p* < 0.001), tended to have obesity (*p* = 0.045), had a higher risk of developing dyslipidemia (*p* = 0.041), and higher AIP and CRI-II (*p* < 0.05) levels while having similar low HDL-C and hypertriglyceridemia frequencies. In the multivariate model adjusting for age, high AIP (adjusted odds ratio [aOR], 3.37; 95% confidence interval [CI], 1.22–5.34) and high CRI-II (aOR: 3.80; 95% CI: 2.25–6.35) were independent determinants of LVDD.

**Conclusions:** These results highlight the importance of considering atherogenic indices, primarily AIP and CRI-II in the management of T2DM patients. High AIP and high CRI-II could serve as surrogate markers of LVDD, an early cardiovascular manifestation in patients with T2DM.

## 1. Introduction

Diabetes mellitus remains a major public health concern today, imposing a heavy global burden associated with immense societal and healthcare costs, early deaths, and high morbidity [1]. It is expected that the global incidence, prevalence, deaths, and disability-adjusted life years associated with diabetes will increase significantly in the coming years [2].

The major cause of death among diabetics is cardiovascular disease (CVD), which is linked in part to the development of a specific myocardial dysfunction known as diabetic cardiomyopathy [3, 4]. Thus, a thorough understanding of the early cardiovascular manifestations of diabetes mellitus is a constant concern of international research and prevention programs.

Van den Hurk [5] made the initial discovery of left ventricular diastolic dysfunction (LVDD) in patients with type 2 diabetes mellitus (T2DM) without coronary artery disease or clinical signs of heart failure in the early 1990s. LVDD, particularly in its grade I, which is actually an impaired left ventricular (LV) relaxation, is identified as the first functional impairment during diabetic cardiomyopathy [5, 6] and has an established prognostic value [7].

In patients with T2DM, LVDD has an unclear pathophysiology. Many underlying mechanisms, such as dyslipidemia, hyperglycemia, and inflammation, significantly contribute to the generation of reactive oxygen species or nitrogen species, which are implicated in this process [8].

Daneii et al.'s recent review has demonstrated that the lipid profile and metabolism are as important in the pathogenesis of LVDD as they are in other CVDs [9].

There is evidence that lipid ratios predict cardiovascular risk more accurately than individual lipids [10–12]. Khedr et al. showed that lipid ratios are predictors of LVDD in adolescents with T1DM [13]. To the best of our knowledge, it is unknown if this also applies to adults with T2DM. Thus, the present study is aimed at determining the correlation between serum lipid parameters and atherogenic indices with LVDD in adult patients with T2DM.

## 2. Materials and Methods

### 2.1. Study Design and Patients

In this hospital-based cross-sectional study, we enrolled 203 T2DM outpatients from eight hospitals in the city of Kinshasa, Democratic Republic of Congo (DRC), between March and June 2022.

### 2.2. Inclusion and Exclusion Criteria

All patients who visited the Department of Medicine and Endocrinology's outpatient clinics throughout the predetermined study period were enrolled in the study and provided written informed consent. The inclusion criteria were apparently healthy patients aged above 18 years with T2DM. The exclusion criteria were Type 1 diabetes, pregnancy, lactation, established valvular heart disease, dilated left ventricle, elevated filling pressures, arrhythmias, other comorbidities (chronic obstructive pulmonary disease, pre-existing renal disease, thyroid disorders, or any other pre-existing heart conditions), poor window on transthoracic echocardiography (TTE), and cholesterol-lowering drug use.

### 2.3. Participant Selection

**Figure 1:**
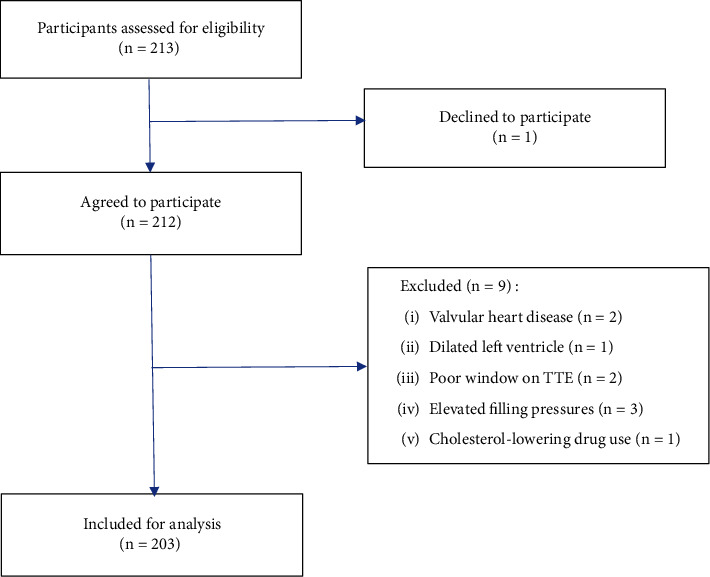
Study flow chart.

At the initial stage of the study, 213 patients were included; of these 213 patients, one chose not to participate and nine were excluded for the following reasons: valvular heart disease (*n* = 2), dilated left ventricle (*n* = 1), poor window on TTE (*n* = 2), elevated filling pressures (*n* = 3), and cholesterol-lowering drug use (*n* = 1). Finally, 203 participants were analyzed.  Figure 1 summarizes the selection procedure.

### 2.4. Independent Variables

Independent variables consisted of the following binary variables: age > 55 years, total obesity, abdominal obesity, hypertension, cigarette smoking, excess alcohol intake, hyperuricemia, dyslipidemia, high total cholesterol (TC), high LDL-C, low HDL-C, hypertriglyceridemia, high atherogenic index of plasma (AIP), high Castelli Risk Index I (CRI-I), high Castelli Risk Index II (CRI-II), high atherogenic coefficient (AC), and high NHC.

The various atherogenic indices were calculated using the following established formulas [14–17]: AIP = Log (TG/HDL‐C), CRI‐I = TC/HDL‐C, CRI‐II = LDL/HDL‐C, AC = (TC–HDLc)/HDL‐C, and NHC = TC‐HDL‐C.

### 2.5. Dependent Variables

LVDD was the dependent variable of the study.

### 2.6. Study Procedures

#### 2.6.1. Medical History

A standard questionnaire was used to collect medical history data, which focused on self-reported age and gender, excessive alcohol consumption, cigarette smoking habits, family history of diabetes and hypertension, and current use of medications for chronic illnesses, particularly oral contraceptives, statins, antiplatelet agents, hypouricemics, and hormone replacement therapy. Patient history of any cardiovascular incidents, such as cardiovascular surgery, ischemic heart disease, heart failure, chronic renal disease, and ischemic stroke, was also recorded.

#### 2.6.2. Anthropometric Data

A final-year medical student who had been trained for this task measured the anthropometric parameters.

#### 2.6.3. Laboratory Measurements

Blood samples obtained in the morning between 8 : 00 AM and 10 : 00 AM after a 10–12 h overnight fast were used for all analyses performed in the same laboratory using the standard methods detailed elsewhere [18].

#### 2.6.4. Echocardiographic Data

Each patient underwent a thorough two-dimensional TTE by two certified cardiac sonographers using commercial 3.5 MHz equipment (Vivid T8, GE Health Care, Freiburg, Germany). LV diastolic function was evaluated in accordance with the 2016 updated guidelines of the American Society of Echocardiography (ASE) and the European Association of Cardiovascular Imaging (EACVI) [19]. In the apical four-chamber view, various LV diastolic function parameters were recorded. According to the 2016 ASE/EACVI recommendations, in patients with a preserved LV ejection fraction (≥ 50%) and no structural heart disease, LV diastolic function was graded as normal or LVDD, using parameters *E*/*e*′, *e*′ velocity (septal and/or lateral), tricuspid regurgitation peak velocity, and maximal left atrial volume indexed for body surface area that were measured. Pulsed Doppler was used to record the mitral E and A waves. At the lateral mitral annulus, *e*′ was measured.

### 2.7. Operational Definitions

T2DM was defined on the basis of the following criteria: fasting glycemia ≥ 7 mmol/L and/or a glycated hemoglobin ≥ 6.5% and/or a personal known history of diabetes mellitus controlled with diet or an oral antidiabetic agent, as per the Consensus Report from the American Diabetes Association (ADA) and European Association for the Study of Diabetes [20].

T1DM diagnosis was considered in insulin-dependent patients with plasma Cpeptide020mmol/L [21].

In this study, LVDD refers specifically to impaired relaxation or LVDD grade I, defined as per the latest guidelines of the ASE/EACVI 2016 algorithm, whereas elevated filling pressures are referred to as LVDD of grade II and higher.

Dyslipidemia was classified as having an HDL-C level of less than 1.03 mmol/L for males and less than 1.04 mmol/L for females, as well as an LDL-C level of more than 3.38 mmol/L, a TC level of more than 5.17 mmol/L, and a triglyceride (TG) level of more than 1.69 mmol/L [22].

High levels of CRI-I, CRI-II, AC, AIP, and NHC were defined by values of these indices above the 75th percentile.

A fasting uric acid level greater than 420 mmol/L was considered hyperuricemia [23].

WC greater than 94 cm for men and larger than 80 cm for women was used to identify abdominal obesity. A BMI of 30 kg/m^2^ or more was considered total obesity.

### 2.8. Statistical Analysis

Results are summarized as counts and percentages for qualitative variables and as mean ± standard deviation (SD) for quantitative variables. Fischer's exact or Pearson's chi-square tests were employed to compare the proportions. To compare the means of two groups with normal distributions, Student's *t*-test was used. The Mann–Whitney's *U* test was applied in cases of skewed distributions to compare the medians. The Kolmogorov–Smirnov test was used to evaluate whether each variable had a normal distribution.

Cross-tabulations and analysis of binary variables (including age > 55 years, total obesity, abdominal obesity, hypertension, cigarette smoking, excess alcohol intake, hyperuricemia, dyslipidemia, high TC, low HDL-C, hypertriglyceridemia, high AIP, high CRI-I, high CRI-II, high AC, and high NHC) were performed to select variables for multivariable analysis. The variables that had a *p* value of < 0.25 in the bivariable analysis were selected as candidates for multivariable analysis. Ultimately, a multivariable logistic regression analysis was conducted using backward selection, and variables with a *p* value below 0.05 were considered statistically significant determinants of LVDD.

The odds ratios (ORs) and their 95% confidence intervals (CIs) were calculated to assess the degree of association between the variables and LVDD. When associations were observed between LVDD and these independent variables, the interactions were tested to highlight potential age-modifying effects in a conditional logistic regression. All data were analyzed using the Statistical Package for the Social Sciences (version 24; IBM Corp., Armonk, NY).

### 2.9. Ethical Considerations

The Helsinki Declaration of 1964 was strictly followed in the conduct of this study. The National Health Ethics Committee (No. 219/CNES/BN/PMMF/220) approved this study. We certify that no artificial intelligence or large language model was used at any stage of this research manuscript writing [24].

## 3. Results

### 3.1. General Characteristics of the Participants

A total of 203 patients (111 men and 92 women) with T2DM were included in the analysis, with a sex ratio of 1 : 2. The average age of the patients was 59.9 ± 13.6 years.

### 3.2. Frequency of LVDD

**Figure 2:**
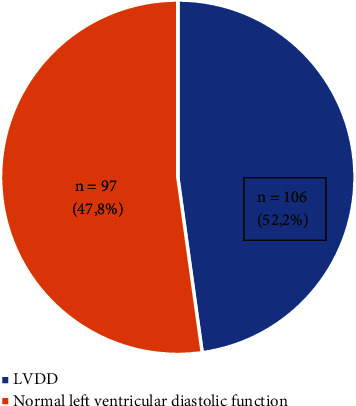
Frequency of LVDD.

Figure 2 depicts that 97 patients (47.8%) had LVDD.

### 3.3. Demographic, Clinical, and Biological Characteristics According to the Diastolic Function

Table 1 illustrates that at comparable weight, height, and WC, patients with LVDD were significantly older and had higher SBP, MBP, and PP. Additionally, at similar levels of creatinine, blood glucose, HbA1C, CT, HDL-C, LDL-C, and TG, those with LVDD had higher AIP and CRI-II than those without LVDD.

### 3.4. Participants' Cardiovascular Risk Factors Based on Diastolic Function

As shown in Table 2, 140 (69.0%) patients had dyslipidemia, which was significantly more common in patients with LVDD. Patients with LVDD were more often over 55 years old (*p* < 0.001) and tended to have obesity (*p* = 0.045) compared with those without LVDD. Furthermore, they more often had dyslipidemia (*p* = 0.041), high LDL-C (*p* = 0.028), high AIP, and high CRI-II than those without LVDD for similar frequencies of low HDL-C levels and hypertriglyceridemia.

### 3.5. Determinants of Diastolic Dysfunction

Table 3 shows the results of the univariate and multivariate analyses. In univariate analysis, age>55 years, hypertension, abdominal obesity, high LDL-C, high AIP, and significantly high CRI-II were associated with LVDD. Following age adjustment, among patients under 55 years old, high LDL-C, high AIP, and high CRI-II were associated with LVDD. On the other hand, among patients over 55 years of age, even after adjusting for age, high AIP and high CRI-II were identified as independent determinants of LVDD.

## 4. Discussion

This study is aimed at determining the relationship between serum lipid parameters and atherogenic indices with LVDD in patients with T2DM. Almost half of patients had LVDD. High AIP (aOR: 3.37; 95% CI: 1.22–5.34) and high CRI-II (aOR: 3.80; 95% CI: 2.25–6.35) were independently associated with LVDD.

The systematic meta-analysis of Bouthoorn et al. reported in-hospital LVDD prevalence in patients with diabetes ranging from 19% to 81% [25]. In the present study, its prevalence was 47.8%, which is close to that found by other authors [26–28] and to the pooled prevalence (48%) reported in the above-mentioned meta-analysis [25]. This wide disparity in the frequencies of LVDD could be explained by various factors, such as the profile of the patients, mainly their comorbidities [29] and their lifestyle [30–32].

Patients with LVDD were significantly older, mostly > 55 years, which was associated with a fourfold increase in the risk of LVDD. This finding is in accordance with previous studies that showed a predominant influence of aging on diastolic function [26, 33–35]. The underlying causes may be age-related anomalies of the myocardial structure and microvasculature, such as increased fibrosis, cardiomyocyte hypertrophy, and decreased microvascular density [36, 37]. Klotho deficiency associated with aging has recently been proposed as a new mechanism for the occurrence of these abnormalities [38].

Our results indicated that hypertension was significantly associated with LVDD, which was consistent with the findings of Jiang et al. [39] who found that hypertension in patients with diabetes is significantly related to LVDD. However, in the current investigation, this association was no longer significant after adjusting for age.

Lipid profile and metabolism are as important for the pathogenesis of LVDD as they are for other cardiovascular conditions [9]. However, there is no typical lipid profile in patients with LVDD. Participants in this study, with, and without LVDD, possessed comparable levels of TG, LDL-C, HDL-C, and CT; however, they had higher AIP and CRI-II than those without LVDD. This brings to mind the best predictive value of lipid ratios relative to individual lipid profiles as individual measurements [40, 41].

In the present study, high LDL-C was the only dyslipidemia that was associated with LVDD, but this association was not significant after adjusting for age. On the other hand, two atherogenic indices, high AIP and high CRI-II, were associated with LVDD. This association remained even after adjusting for age, with the risk of LVDD increasing threefold and fourfold, respectively.

This result differs from that of Khedr et al. [13], who demonstrated a correlation between low HDL-C and LVDD (and not CRI-II), but it is comparable to the correlation between TG/HDL-C and LVDD that was also demonstrated by those same authors [13]. Clinicians and researchers frequently employ individual lipid profiles as single measurements of CVD risk factors [41]. The distribution of lipid particle sizes is reflected by AIP, which accounts for the balance between harmful and protective lipids and is significantly correlated with other atherosclerosis risk factors [42]. AIP has also been demonstrated to be a more accurate risk predictor than individual lipid profiles [39, 40], making it a potent proxy for the CVD risk. Its association with LVDD has been demonstrated in young people with T1DM [13]. Our study is the first to show this association among adults with T2DM.

CRI-II, which is the ratio LDL-C to HDL-C, has demonstrated predictive value. Indeed, it has been associated to increased carotid intima-media thickness risk [42], to myocardial infarction [43, 44], and is considered a better predictor of the severity of cardiac atherosclerotic coronary artery disease, compared to LDL-C or HDL-C [45]. Furthermore, Wang et al. [46] demonstrated an independent positive association of nontraditional lipid profiles, including the LDL-C/HDL-C ratio, with concentric LV hypertrophy, a hallmark of preclinical CVD, in the general population of rural China.

To the best of our knowledge, our study is the first to demonstrate the independent correlation between CRI-II and LVDD in individuals with T2DM.

Patients with LVDD were more likely to be obese than those without LVDD. This is in line with earlier studies that found a link between obesity and a higher risk of LVDD [47, 48], even among metabolically healthy individuals [49]. A recent update on the molecular mechanisms involved in this link [50] points to a modified expression of proinflammatory cytokines, adipokines, and hormones, which activate pathological processes such as oxidative stress and inflammation in myocytes. Another factor is the activation of the renin-angiotensin-aldosterone system, which increases inflammation and leads to structural remodeling, thereby resulting in LVDD-causing heart injury.

### 4.1. Clinical Implications

This study brings a new perspective to an existing body of research on the practical importance of the AIP and CRI-II. Indeed, studies have shown that AIP is a strong predictor of the likelihood of developing coronary artery disease in individuals with prediabetes [51] and T2DM [51, 52]. In addition, the Nagasaki Islands study [53] revealed that diabetic patients with high AIP had a significant risk of progression of carotid intima-media thickness and increased arterial stiffness. Moreover, AIP has been shown to predict the risk of major adverse cardiovascular events in T2DM patients [54]. Furthermore, AIP has been shown to be positively associated with the frequency and severity of microvascular complications of diabetes [55]. Not to mention, a 4-year period observational cohort study of 2356 patients who underwent percutaneous coronary intervention [56] found that AIP is linked to major cardiovascular and cerebrovascular adverse events after percutaneous coronary intervention in T2DM patients. The clinical significance of CRI-II has also been extensively proven [42–46]. However, our study is the first to establish its independent correlation with LVDD in adult patients with T2DM. This current study uncovers for the first time another potential use of AIP and CRI-II as surrogate markers for subclinical LVDD, one of the first signs of myocardial involvement in adult patients with T2DM [57] and an important predictor of heart failure [58]. The latest heart failure guidelines [59] focus on detecting these asymptomatic changes of LV function early and pinpointing its primary risk factors. Therefore, earlier detection and management of LVDD using AIP and CRI-II as surrogate markers in T2DM patients may help prevent or delay the onset of chronic heart failure. Indeed, the CVD continuum in T2DM emphasizes the potential for treatment at each stage to help avoid or delay the onset of symptomatic heart failure [60]. Interventional studies are needed to determine whether it would be beneficial to provide additional treatments like inhibitors of sodium-glucose co-transporter-2 (SGLT2), along with lipid-lowering medications, to those with high AIP and CRI-II, since empagliflozine has been demonstrated to enhance diastolic function in a genetic model of T2DM ob/ob mice [61].

### 4.2. Study Strengths and Limitations

To the best of our knowledge, the present study is the first to demonstrate this correlation of AIP and CRI-II with LVDD in adults with T2DM. We herein evaluated the diastolic function using Doppler echocardiography, which is available, inexpensive, and noninvasive [62, 63]. However, it would be necessary to integrate other parameters, notably those emerging from the research domain, such as speckle-tracking strain [63, 64], to improve TTE diagnosis accuracy. This analysis was conducted on a relatively small sample of patients with diabetes (*n* = 203). A larger study should be considered in the future.

## 5. Conclusions

LDL-C may be a useful surrogate marker of LVDD in adult T2DM in the same way as AIP. These results highlight the need to consider not only lipid parameters but also atherogenic indices, primarily LDL-C, and AIP, to suspect the existence of LVDD, an early cardiovascular manifestation in patients with T2DM.

Further studies with a larger sample size are required to confirm our findings. They should be prospective and population-based and should use serial imaging and blood measurement of lipid parameters to identify a possible causal link that cannot be confirmed by a cross-sectional study. Furthermore, a better understanding of the influence of AIP in particular, and that of other atherogenic indices and lipid parameters in general, on structural and functional abnormalities of the left ventricle requires additional studies.

## Figures and Tables

**Table 1:** Demographic, clinical, biological, and ultrasound characteristics according to the diastolic function.

**Variables**	**Overall** **N** = 203	**LVDD** **n** = 97	**Normal diastolic function** **n** = 106	**p**
Age (years)	59.9 ± 13.6	63.9 ± 10	56.4 ± 13.3	< 0.001
Sex, male, *n* (%)	111 (54.7)	56 (57.7)	55 (51.9)	0.244
Weight (kg)	83.4 ± 16.8	84.9 ± 17.5	82.1 ± 16.1	0.239
Height (cm)	170.5 ± 9.8	171.6 ± 7.5	169.5 ± 11.5	0.132
BMI (kg/m^2^)	28.6 ± 5.2	28.7 ± 5.1	28.9 ± 8.8	0.865
Waist (cm)	104.2 ± 21.4	101.7 ± 6.8	106.4 ± 29.4	0.690
SBP (mmHg)	143.6 ± 17.8	147.1 ± 18.7	139.9 ± 16.0	0.004
DBP (mmHg)	79.1 ± 10.5	78.5 ± 10.6	79.7 ± 10.3	0.388
MBP (mmHg)	100.6 ± 10.5	102.2 ± 10.6	98.9 ± 10.2	0.027
PP (mmHg)	64.5 ± 17.7	67.3 ± 18.5	61.4 ± 16.2	0.017
Creatinine (mmol/L)	78.0 (76.0–85.5)	76.0 (67.0–88.5)	88.0 (78.0–95.0)	0.082
FBG (mmol/L)	8.7 ± 2.7	8.7 ± 2.7	8.7 ± 2.7	0.929
HbA1C (%)	8.0 (7.8–8.5)	8.1 (7.7–9.1)	8.1 (7.6–8.8)	0.510
TC (mmol/L)	4.8 ± 1.3	4.7 ± 1.4	4.9 ± 1.2	0.249
HDL-C (mmol/L)	1.44 ± 0.52	1.4 ± 0.5	1.5 ± 0.6	0.143
LDL-C (mmol/L)	3.21 ± 0.93	3.1 ± 0.9	3.3 ± 1.0	0.162
TG (mmol/L)	1.61 ± 1.07	1.6 ± 1.1	1.7 ± 1.0	0.517
AIP	1.0 (0.9–1.1)	1.2 (1.1–1.4)	0.8 (0.7–0.9)	0.027
CRI-I	3.2 (2.9–3.4)	3.4 (3.0–3.6)	2.9 (2.8–3.4)	0.181
CRI-II	2.2 (1.9–2.4)	2.5 (2.4–2.7)	1.8 (1.6–1.9)	0.001
AC	2.2 (1.9–2.4)	2.4 (2.0–2.6)	1.9 (1.8–2.4)	0.173
NHC	3.3 (3.1–3.5)	3.4 (3.2–3.9)	3.2 (2.8–3.5)	0.136

Abbreviations: AC = atherogenic coefficient, AIP = atherogenic index of plasma, BMI = body mass index, CRI-I = Castelli Risk Index I, CRI-II = Castelli Risk Index II, DBP = diastolic blood pressure, FBG = fasting blood glucose, HbA1C = glycated hemoglobin, HDL-C = high-density lipoprotein cholesterol, LDL-C = low-density lipoprotein cholesterol, LVDD = left ventricular diastolic dysfunction, MBP = mean blood pressure, NHC = non-high-density lipoprotein cholesterol, PP = pulse pressure, SBP = systolic blood pressure, TC = total cholesterol, TG = triglycerides.

**Table 2:** Participants' cardiovascular risk factors based on diastolic function.

**Variables**	**Overall** **N** = 203	**LVDD** **N** = 97	**Normal diastolic function** **N** = 106	**p**
Age > 55 years	140 (69.0)	45 (46.4)	18 (17.0)	< 0.001
Overweight	95 (46.8)	46 (47.4)	49 (46.2)	0.488
Total obesity	62 (30.5)	31 (32.0)	31 (29.2)	0.395
Abdominal obesity	12 (80.0)	6 (85.7)	6 (75.0)	0.045
Hypertension	162 (79.8)	26 (26.8)	15 (14.2)	0.019
Cigarette smoking	19 (9.4)	12 (12.4)	7 (6.6)	0.121
Excess alcohol intake	72 (35.5)	32 (33.0)	40 (37.7)	0.288
Hyperuricemia	34 (16.7)	17 (17.5)	17 (16.0)	0.461
Dyslipidemia	140 (69.0)	76 (78.3)	64 (66)	0.041
High TC	89 (43.8)	41 (42.3)	48 (45.3)	0.386
Low HDL-C	2 (1.0)	1 (1.0)	1 (0.9)	0.729
High LDL-C	75 (36.9)	45 (46.4)	30 (28.3)	0.028
Hypertriglyceridemia	69 (34.0)	30 (30.9)	39 (36.8)	0.232
High AIP	50 (24.6)	39 (40.2)	11 (10.4)	0.001
High CRI-I	50 (24.6)	28 (28.9)	22 (20.8)	0.085
High CRI-II	50 (24.6)	29 (29.9)	21 (19.8)	0.018
High AC	36 (17.7)	17 (23.3)	19 (24.7)	0.497
High NHC	101 (49.8)	45 (47.4)	56 (52.8)	0.264

Abbreviations: AC = atherogenic coefficient, AIP = atherogenic index of plasma, CRI-I = Castelli Risk Index I, CRI-II = Castelli Risk Index II, HDL-C = high-density lipoprotein cholesterol, LDL-C = low-density lipoprotein cholesterol, LVDD = left ventricular diastolic dysfunction, NHC = non-high-density lipoprotein cholesterol, TC = total cholesterol, TG = triglycerides.

**Table 3:** Correlations of hypertension, abdominal obesity, high LDL-C, high AIP, and high CRI-II with LVDD, adjusted for covariables^a^ and stratified by age.

**Variable**	**Univariate analysis**		**Adjusted** **a** **g** **e** ≤ 55**years**		**Adjusted** **a** **g** **e** > 55**years**	
	**p**	**OR (95% CI)**	**p**	**aOR (95% CI)**	**p**	**aOR (95% CI)**
Age > 55 years						
No		1	—	—	—	—
Yes	< 0.001	3.64 (1.36–7.49)	—	—	—	—
Hypertension						
No				1		1
Yes	< 0.001	4.63 (1.92–6.86)	0.280	1.66 (0.45–1.71)	0.507	1.44 (0.49–4.24)
Abdominal obesity						
No		1		1		1
Yes	0.041	1.97 (1.04–3.58)	0.935	1.07 (0.20–1.64)	0.300	0.62 (0.23–1.53)
Hyper LDL-C						
No		1		1		1
Yes	0.024	3.14 (1.66–6.98)	0.036	3.17 (1.92–4.82)	0.224	1.77 (0.71–4.41)
High AIP						
No		1		1		1
Yes	0.032	2.92 (1.46–3.94)	0.001	3.67 (2.06–7.78)	0.019	3.37 (1.22–5.34)
High CRI-II						
No		1		1		1
Yes	0.039	2.06 (1.13–3.34)	0.012	3.92 (2.67–5.95)	< 0.001	3.80 (2.25–6.35)

Abbreviations: AIP = atherogenic index of plasma, CRI-II = Castelli Risk Index II, LDL-C = low-density lipoprotein.

^a^Covariables: total obesity, cigarette smoking, excess alcohol intake, hyperuricemia, dyslipidemia, high TC, low HDL-C, hypertriglyceridemia, high CRI-I, high AC, and high NHC.

## Data Availability

Although the consent given by the study participants did not include a clause relating to data sharing with third parties, anonymized data may be made available to investigators for analysis upon reasonable request to the corresponding author.
